# Jacobi on Infant Diet

**Published:** 1874-10

**Authors:** 


INFANT DIET. By A. Jacobi, M.D., Clinical Professor of Diseases of Children, College of Physicians and Surgeons, New York. Revised, enlarged and adapted to popular use by Mary Putnam Jacobi, M.D. New York: G. P. Putnam's Sons, 1874. 12 mo., cloth, pp. 119. Price, 75c.

The little volume before us is designed to enlighten the minds of mothers upon the *vitally* important subject of infant diet. We say vitally important, because we firmly believe that an enlightened and general attention to this subject would eventually lead to an improved dietetic management which, in our cities, would rob the grave of a very large percentage of its infantile victims. Whilst the volume given us by Mrs. Jacobi is designed for popular reading, it will be found profitable reading likewise for the medical profession.

---

A FREE AND INDEPENDENT TRANSLATION of the first and fourth books of the ÆNEID OF VIRGIL: wherein are unfolded the travels of Æneas, the origin of the Roman Empire, the stratagems employed by the goddess Juno (happily without success) to nip that important enterprise in the bud, the counterplots of the goddess Venus and her mischievous little son Cupid, and the furious love and romantic death of queen Dido. In hexameter and pentameter; with illustrations by Thomas Worth. Pamphlet, pp. 22. Price 25c.

Those who relish good *classical laughter* will likely be repaid by sending a quarter to Herald office, Winsted, Conn., for the above.

